# Effectiveness of smart health-based rehabilitation on patients with poststroke dysphagia: A brief research report

**DOI:** 10.3389/fneur.2022.1110067

**Published:** 2023-01-09

**Authors:** Jian-Rong Zhang, Yu-E Wu, Yan-Fang Huang, Shu-Qing Zhang, Wen-Li Pan, Jin-Xia Huang, Qing-Ping Huang

**Affiliations:** Department of Nursing, Dongguan Houjie Hospital Affiliated to Guangdong Medical University, Dongguan, Guangdong, China

**Keywords:** poststroke dysphagia, smart health, rehabilitation, nursing, rehabilitation nurse

## Abstract

**Objective:**

This study aimed to evaluate the effectiveness of smart health-based rehabilitation on patients with poststroke dysphagia (PSD).

**Methods:**

We recruited 60 PSD patients and randomly allocated them to the intervention (*n* = 30) and control (*n* = 30) groups. The former received the smart health-based rehabilitation for 12 weeks, whereas the latter received routine rehabilitation. Water swallow test (WST), standardized swallowing assessment (SSA), swallow quality-of-life questionnaire (SWAL-QOL), stroke self-efficacy questionnaire (SSEQ), perceived social support scale (PSSS) and nutritional measurements including body weight, triceps skinfold thickness (TSF), total protein (TP), serum albumin (ALB) and serum prealbumin (PA) in both groups were measured.

**Results:**

When the baseline WST, SSA, SWAL-QOL, SSEQ, PSSS and nutritional measurements were examined, there was no significant difference between the intervention group and the control group (*P* > 0.05). After rehabilitation interventions, the WST and SSA scores in the intervention group were significantly lower than those in the control group (*P* < 0.01). The SWAL-QOL, SSEQ and PSSS scores in the intervention group were significantly higher than in the control group (*P* < 0.01). Compared with the control group, the intervention group showed an increase in the serum levels of PA (*P* < 0.01). However, no statistically significant difference existed between the intervention group and the control group in terms of body weight, TSF, TP or ALB (*P* > 0.05).

**Conclusions:**

Overall, our data revealed that smart health-based rehabilitation is significantly beneficial to the swallowing function, quality of life, self-efficacy, and social support for PSD patients when compared with routine rehabilitation. However, nutritional measurements were not significantly improved in such patients under the smart health-based rehabilitation when compared the routine rehabilitation. In the future, it is necessary to extend the intervention time to further evaluate the long-term efficacy of smart health-based rehabilitation on nutritional measurements of PSD patients.

## 1. Introduction

Poststroke dysphagia (PSD), or post stroke swallowing difficulty, is one of the most common complications of ischemic stroke patients (1). Impairment in the central nervous system, cortical, or subcortical parts of the brain after stroke can impair swallowing physiology and further leads to true bulbar paralysis (2). According to epidemiological statistics, the incidence rate of PSD in China is 51–73% (3). If PSD is not intervened in time, it further leads to difficulties in eating and drinking, resulting in malnutrition, aspiration pneumonia, dehydration, and asphyxia, which seriously affects the prognosis of PSD patients (4, 5). PSD not only increases the economic burden of patients and prolongs their hospitalization time, but also brings a greater care burden to their family members (6, 7). Furthermore, PSD patients are prone to mental and psychological problems. For example, major and minor depression occur in 20–65% of PSD patients (8–11). Moreover, studies show that the presence of PSD is associated with anxiety, bringing individual psychosocial consequences such as fear, and frustration (12, 13). At the same time, PSD is one of the key factors causing disability and death of patients (14).

Currently, the conventional swallowing interventions include transcutaneous neuromuscular electrical stimulation (NMES), biofeedback and lingual strength training (15, 16). However, in China, the limited coverage of medical insurance for PSD patients' rehabilitation programs has brought a greater economic burden to PSD patients and their families (17). Since the implementation of the Diagnosis Related Groups (DRG) payment system in China, most PSD patients must transition from inpatient rehabilitation to home-based rehabilitation due to the limitation of the average hospital stay (18). However, China's community rehabilitation medical resources are in relative shortage, especially professional physiotherapists in the community. This leads to the lack of professional home-based rehabilitation guidance for discharged PSD patients, making the rehabilitation effect of most discharged PSD patients unsatisfactory (19).

To date, smart health devices including smartphones, patient-monitoring devices and wireless devices have enabled patients and health care providers to use health information anytime, anywhere by adding modern technology to health care management systems (20). Smart health employs a variety of different features, including smartphone-based applications. China has now exceeded 500 million smartphone users (21). The WeChat smartphone application has been gradually applied to the field of medical rehabilitation in China because it is convenient and fast, with the advantages of quickly sending messages, video, voice, and pictures through the network, supporting multi-group chat and realizing information sharing (22). The use of smart health makes it possible to combine information technology with individualized continuous rehabilitation.

For PSD patients in the rehabilitation period after discharge, home rehabilitation is a long-term process. WeChat's characteristics of interactivity, immediacy and wide area also allow for expanding the continuity of PSD home-based rehabilitation (23). Specifically, home-based rehabilitation exercise videos on a WeChat public account can be watched repeatedly, which is convenient for PSD patients and their caregivers for learning. In WeChat synchronized videos, PSD caregivers can complete the basic rehabilitation training of swallowing function and diet modification for PSD patients under the supervision of physiotherapists, nutritionists and rehabilitation nurses. WeChat groups can also help realize information sharing between health providers and PSD patients, among health provider team members, and even between peer educators and PSD patients (24).

To date, WeChat-based rehabilitation programs have been widely used in several chronic diseases, including for diabetes patients (25), postoperative women with breast cancer (26), and patients with chronic obstructive pulmonary disease (27). However, very few WeChat-based rehabilitation programs have been developed to support PSD patients. Li et al. (28) explored the effects of smart health-based rehabilitation programs on middle-aged stroke patients. However, that study did not focus on PSD patients, and self-efficacy, social support and nutritional status were not included in this research. The aim of this study was to explore the effect of smart health-based rehabilitation programs on patients with PSD in terms of swallowing function, quality of life, self-efficacy, social support and nutritional status.

## 2. Materials and methods

This was a prospective, parallel-group, randomized, assessor-blinded clinical pilot trial. This clinical trial was approved by the hospital ethics committee, in line with the *Declaration of Helsinki*, and all patients signed informed consent. This study has been registered in the open science framework (OSF) clinical trial registry (Registration number: DOI https://doi.org/10.17605/OSF.IO/T4UDE).

### 2.1. Participants, selection criteria and randomization

Participants were selected using the following inclusion criteria: (1) age between 40 and 80 years old; (2) first-time stroke was screened through neurological examination by the attending neurologist and confirmed by computed tomography or magnetic resonance imaging findings according to the WHO definition (29); (3) dysphagia following stroke was confirmed by a videofluoroscopic swallowing study (VFSS). Additionally, to control for spontaneous recovery, enrolment into the study had to occur at least 4 weeks after PSD onset. The exclusion criteria were cognitive impairment or severe communication disorders, serious psychologic disorders including major depressive disorder, bipolar disorder, schizophrenia, *etc*., known history of dysphagia prior to the stroke [(a) other neurological conditions that may explain dysphagia: amyotrophic lateral sclerosis, multiple sclerosis, or Parkinson's disease; (b) swallowing disorders caused by surgery or radiotherapy applied to the head and neck region; (c) dysphagia due to drug toxicity], unstable cardiopulmonary status, head and neck cancer, implanted cardiac pacemaker, nasogastric tube, and history of seizures or epilepsy. Random numbers were generated by the center computer. The random numbers were placed in opaque and sealed envelopes, and PSD patients were randomly assigned to the control group or intervention group in a ratio of 1:1 according to the random allocation order.

### 2.2. Treatment methods

#### 2.2.1. Control group

According to the guidelines of the European Stroke Organization and European Society for Swallowing Disorders in 2021 (30), the 12-week routine rehabilitation was patient-oriented and adjusted based on PSD patient swallowing function *via* VFSS (31). (1) Oral–facial and laryngeal motor exercises according to the stroke rehabilitation and recovery guideline. (2) Sensorial stimulation of the oral cavity. These sensory stimulation methods were patient-oriented and customized to the patients' swallowing abilities. (3) Diet modification: diet modification and nutritional support were individualized to match the PSD patients' nutritional and functional statuses under the guidance of registered nutritionists and a nutritional support team. Four weeks after PSD onset, according to the stroke rehabilitation and recovery guideline, dysphagia rehabilitation services were delivered by a multidisciplinary team (MDT) of healthcare providers. According to the American Stroke Association guideline, our MDT team includes neurologists who have specialized training or board certification in rehabilitation medicine, rehabilitation nurses, nutritionists, physiotherapists, and speech and language therapists (32). The MDT held case meetings every week. During the meeting, MDT members shared and discussed the patient's rehabilitation care pathway in combination with data from the clinical swallowing evaluations, the results of rehabilitation strategies, the patient's medical condition and clinical judgment.

#### 2.2.2. Intervention group

PSD patients in the intervention group received smart health-based rehabilitation in addition to the routine rehabilitation. The WeChat cloud platform provided continuous rehabilitation training programs for PSD patients, which were set up for five modules: patient symptom assessment and clinical database establishment, health education, smart health-based oral–facial and laryngeal motor exercises and sensorial stimulation exercises, diet modification and WeChat group. (1) Patient symptom assessment and clinical database establishment: rehabilitation nurses first used the Eating Assessment Tool-10 (EAT-10) tool to identify patients with dysphagia. Patients with an EAT-10 score >3 points underwent the water swallow test (WST) test. The EAT-10, WST results and other clinical data were uploaded to the WeChat cloud platform to realize the tracking of the patients' condition and the sharing of information among MDT members. During the follow-up after discharge, the corresponding data on the WeChat cloud platform was updated in real time and become the reference for the MDT to adjust the rehabilitation plan. (2) Health education: hospital information engineers set up the WeChat public account, and the rehabilitation nurses guided patients to follow the WeChat public account when they were discharged to ensure that every patient could obtain health education information through the online platform. The rehabilitation nurses distributed the information of the PSD follow-up health education manual to patients every 2 days through text, pictures, videos and other forms after the PSD patients were discharged from hospital to strengthen their health education. The rehabilitation nurses set up the WeChat theme interactive sign-in form in the WeChat cloud platform to monitor the reading of PSD patients in real time and reply and answer their questions in time in the cloud platform. (3) Smart health-based oral–facial and laryngeal motor exercises and sensorial stimulation exercises: After discharge from the hospital, the physiotherapists uploaded the sample video of basic swallowing function rehabilitation training to the WeChat cloud platform. The video was prepared by the physiotherapists using electronic products, combined through editing and subtitles. Caregivers of PSD patients could repeatedly watch and learn basic swallowing function rehabilitation skills on the cloud platform at any time to strengthen their proficiency in skill application. In addition, on the terminal of the cloud platform, the physiotherapists supervised and guided the caregivers of PSD patients on tongue muscle training and cheek muscle training (pouting, empty swallowing, etc.) every day in the first week through the video connection of the WeChat cloud platform. In the remaining 11 weeks, the physiotherapists monitored the completion of oral–facial and laryngeal motor exercises and sensory stimulation exercises of PSD patients in real time through the WeChat clock in applet of daily monitoring report of rehabilitation exercises. (4) Diet modification: diet modification was performed on the WeChat MDT platform. First, the nutritionists calculated the calories required by the PSD patients and the proportion of the nutrients according to their medical history, weight and activity ability (data were mainly retrieved from the WeChat cloud platform). On the WeChat MDT platform, the nutritionist informed the nutrition assessment results to the neurologists, and the neurologists issued the food for PSD patients after consultation with the physiotherapists. After discharge from the hospital, the rehabilitation nurses conducted one-to-one online video guidance for three consecutive days based on the WeChat cloud platform. The rehabilitation nurses then conducted examinations with the caregivers of PSD patients once a week through the WeChat cloud platform, and the neurology nurses were responsible for recording them. The records included evaluating the general state and feeding state of the patient before eating, checking the eating environment, state, posture, tableware, food, and eating and feeding methods. The rehabilitation nurses ensured the accuracy of the implementation of the program by the patients' caregivers and answered the different problems encountered by them. The nutritionists conducted weekly video communication through the WeChat cloud platform, re-formulated the food for the next week with the family members, and adjusted the consistency of the food according to the feedback of neurology specialists and physiotherapists on the WeChat MDT platform. (5) WeChat group: the WeChat group included three WeChat subgroups. The first was the WeChat Med subgroup. In this subgroup, in addition to the weekly case meetings of the MDT as in the control group, the MDT members in the WeChat Med subgroup could share real-time information and promote mutual communication and cooperation. The second was the healthcare providers–PSD patients–caregivers of PSD patients WeChat subgroups. In this subgroup, PSD patients could get regular follow-up visits and real-time health guidance online. In the third peer education subgroup, the MDT members invited patients with better disease control and their caregivers to participate and provide social and peer support to PSD patients. All participants received weekly follow-up. After the 12-week smart health-based rehabilitation intervention, the MDT members conducted the final evaluation and follow-up of patients. The follow-up was conducted in the form of a family visit. It included the evaluation of disease status and swallowing function, quality of life, self-efficacy, social support and nutritional status.

### 2.3. Outcome measures

#### 2.3.1. Primary outcomes

(1) *Swallowing function assessments:* ① *WST*. The rehabilitation nurses gave 2–3 teaspoons of water to the patients with PSD and instructed them to drink it once. The WST evaluation criteria were scored by a 5-point Likert scale (33). *Standardized swallowing assessment (SSA)*. The swallowing function of patients in the two groups before and after the intervention was evaluated and divided into three parts, with a total possible score of 46 (34). The lower the score, the better the swallowing function.
(2) *Quality of life, self-efficacy and social support assessment:* *Swallow quality-of-life questionnaire (SWAL-QOL)* (35). The questionnaire consists of 11 dimensions and 44 items. A 5-point Likert scale was adopted, with a total possible score of 220. A higher score means a better quality of life. *Stroke self-efficacy questionnaire (SSEQ)* (36). It includes two dimensions of activity of daily living and self-management efficacy, with a total of 13 items. A 10-point Likert scale was adopted, and the possible score ranged from 0 to 100. Higher scores mean a higher self-efficacy level. *Perceived Social Support Scale (PSSS)*. This scale contains 12 items, and each item uses a 1–7 scoring system. The total score of the scale is the sum of item scores, ranging from 12 to 84. The higher the score, the higher the individual's perceived level of social support.

#### 2.3.2. Secondary outcomes

*Nutritional measurements:* Body weight (kg), triceps skinfold thickness (TSF), biomarker [total protein (TP; g/L), serum albumin (ALB; g/L), serum prealbumin (PA; mg/L)].

### 2.4. Statistical analysis

Statistical product service solutions (SPSS) 19.0 statistical software (SPSS Inc., Chicago, IL, USA) was used for data analysis. Data were presented by means (standard deviation, SD) for continuous variables and frequencies and percentage (%) for categorical variables. The independent *t*-test and Chi-square test were used to compare differences between the two groups at the baseline and 12th week. A value of *p* < 0.05 (two-tailed) indicated statistical significance.

## 3. Results

**Figure 1:**
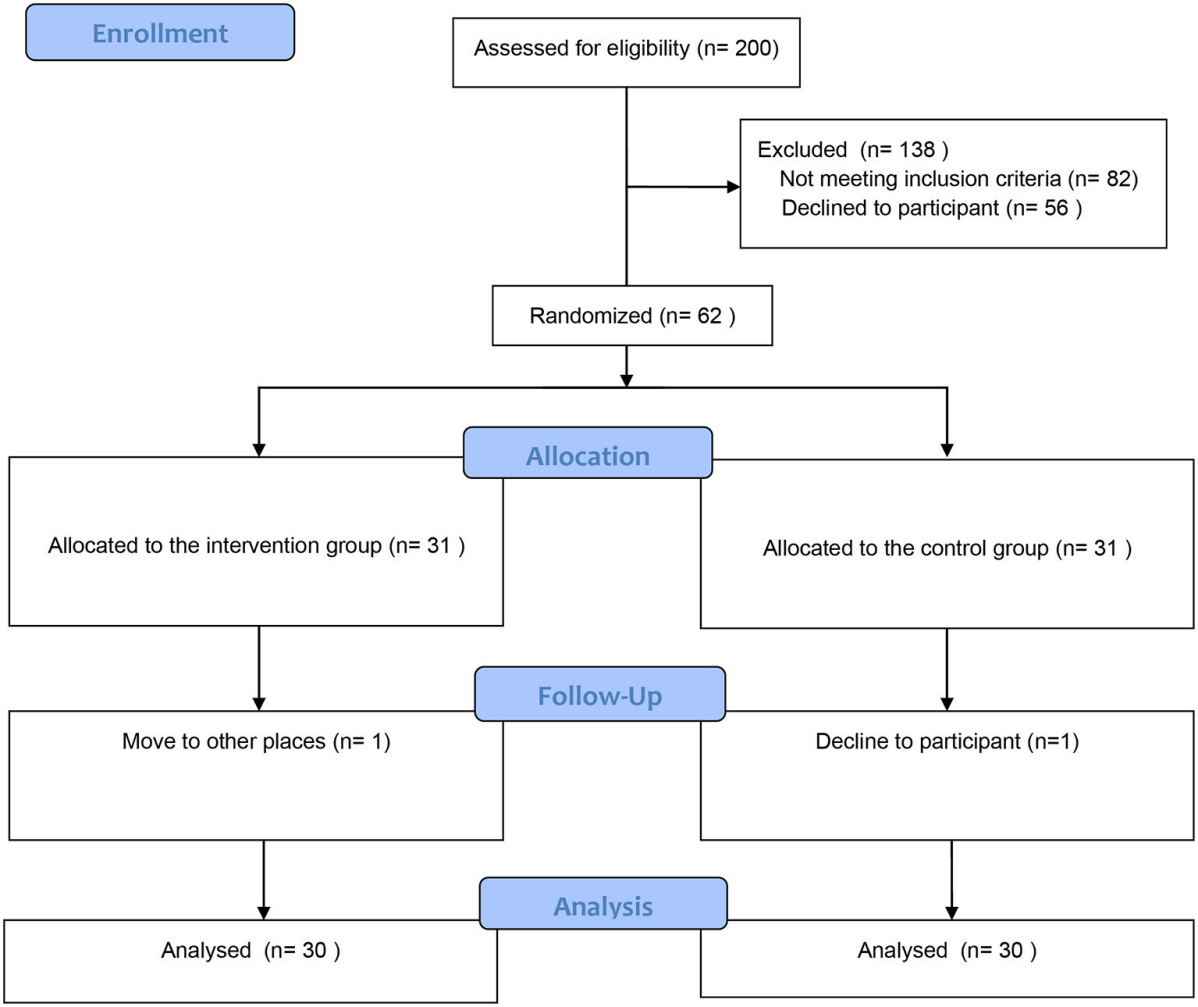
Consort flow diagram.

According to the sample size calculation with the power analysis sample size (PASS) tool (α = 0.05, 1-β = 0.8 and effect size of standardized swallowing assessment score=0.87), a total of 62 patients with PSD were included in this study. From February 2020 to December 2021, 200 participants were screened in this study, and 140 participants were excluded. In the follow-up phase, a participant in the control group was dropped out because he refused to continue to participate; a participant in the intervention group was dropped out because he moved to the other places. Finally, 60 participants (Control group: *n* = 30; Intervention group: *n* = 30) were included for data analysis (Figure 1).

### 3.1. Comparison of baseline data

There were no significant differences in general data such as age, gender, marital status, education level, stroke events, time post-stroke, types of strokes and healthcare insurance between the intervention group and the control group (*p* > 0.05) (Table 1).

**Table 1:** Baseline of study characteristics.

	**Intervention group (*N = 30*)**	**Control group (*N = 30*)**		

**Variables**	**Mean (** ***SD*** **)**	**Mean (** ***SD*** **)**	***t***	***P***
Age (years)	68.10 ± 2.34	69.22 ± 3.26	1.53	0.13
Time post-stroke (weeks)	17.37 ± 2.64	18.09 ± 2.53	1.08	0.29
**Variables**	***N*** **(%)**	***N*** **(%)**	***χ^2^***	***P***
Gender			0.32	0.57
Male	20 (66.67)	22 (73.33)		
Female	10 (33.33)	8 (26.67)		
Marital status			0.58	0.45
Married	27 (90.00)	25 (83.33)		
Single	3 (10.00)	5 (16.67)		
Education level			0.53	0.77
Undergraduate	3 (10.00)	6 (20.00)		
High school	6 (20.00)	1 (3.33)		
Middle school	21(70.00)	23 (76.67)		
Type of stroke			1.18	0.28
Ischemic stroke Haemorrhagic	24 (80.00)	27 (90.00)		
Stroke	6 (20.00)	3 (10.00)		
Healthcare insurance			0.60	0.44
Yes	14 (46.67)	17 (56.67)		
No	16 (53.33)	13 (43.33)		

### 3.2. Comparison of swallowing function between the two groups

**Figure 2:**
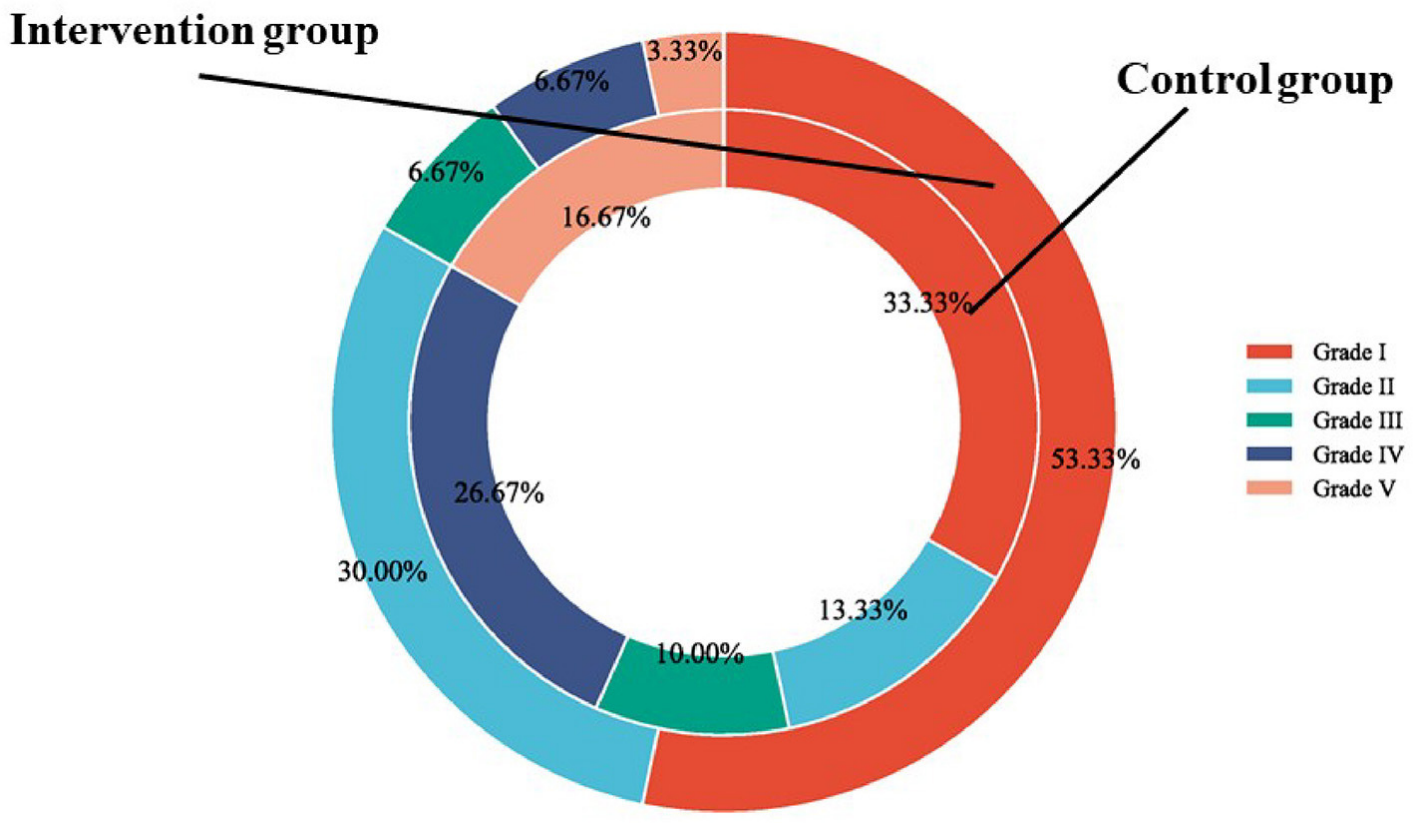
Comparison of WST between the two groups.

**Figure 3:**
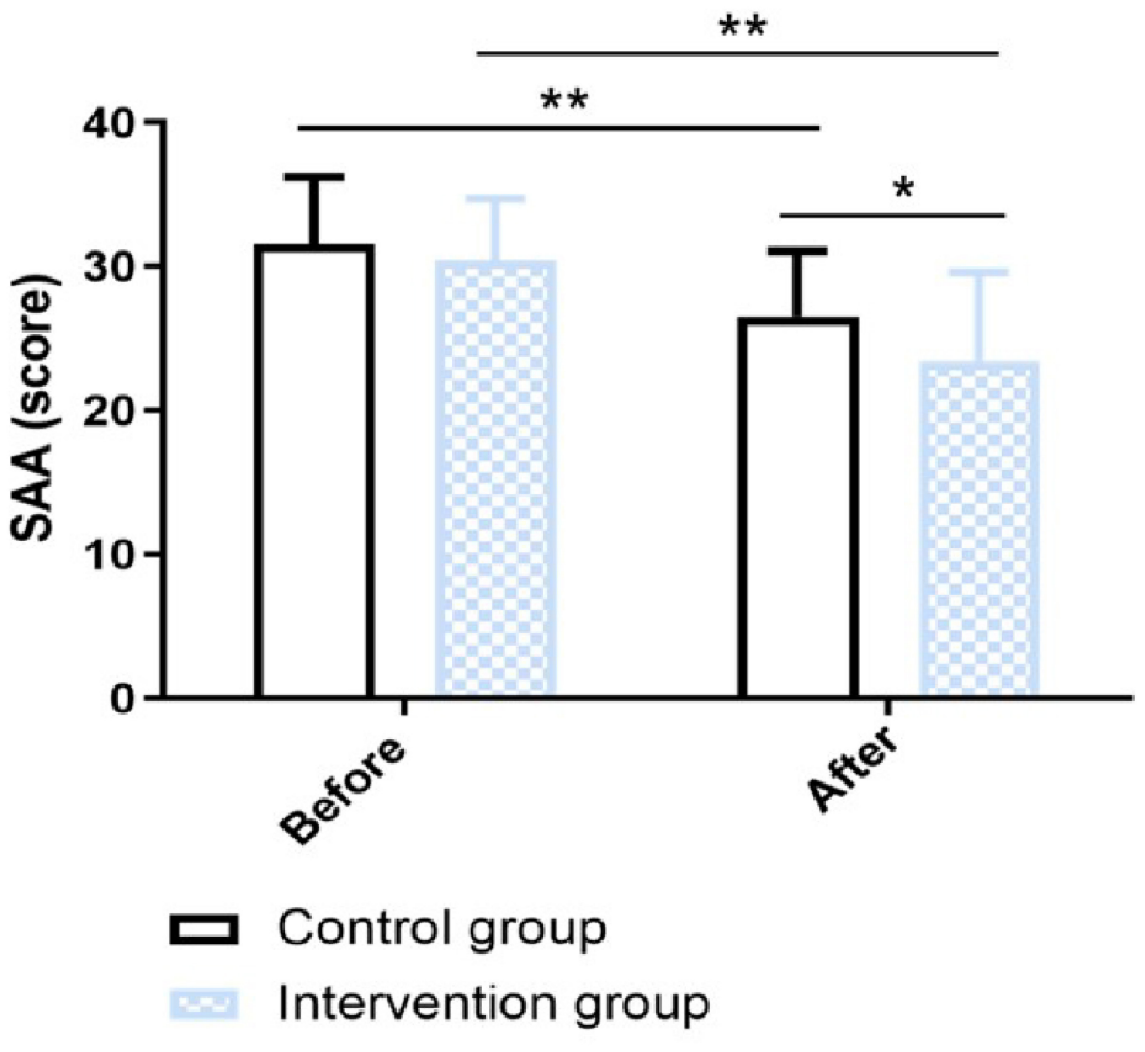
Changes in SAA scores between groups. *P < 0.05, **p < 0.01.

In this research, the swallowing function was measured by WST and SAA score. In terms of the WST, in the intervention group, the proportion of participants in Grade I, Grade II, Grade III, Grade IV, and Grade V was 53.33, 30, 6.67, 6.67, and 3.33%, respectively; In the control group, the proportion of participants in Grade I, Grade II, Grade III, Grade IV, and Grade V was 33.33, 13.33, 10, 26.67, and 16.67%, respectively. There was a significant difference in WST between the two groups (χ^2^ value = 9.77, *P* value = 0.040) (Figure 2). The SAA score was not statistically significantly different between the intervention group and the control group before the intervention (*p* > 0.05). After 12-week intervention, the SAA score of the intervention group was significantly improved than that of the control group (χ^2^ value = 2.20, *P* value = 0.032) (Figure 3).

### 3.3. Comparison of quality of life, self-efficacy and social support between the two groups

SWAL-QOL were used to assess the health-related quality of life, SSEQ was used to assess the self-efficacy, and PSSS was used to assess the social support between the two groups before and after the intervention. Before intervention, there was no significant difference in the quality of life (Supplementary Figure S1A), self-efficacy (Supplementary Figure S1B) and social support (Supplementary Figure S1C) between the two groups (*P* > 0.05). After intervention, the improvement of quality of life (Supplementary Figure S1A), self-efficacy (Supplementary Figure S1B) and social support (Supplementary Figure S1C) was greater in the intervention group than in the control group (*P* < 0.05) (Supplementary Figure S1).

### 3.4. Comparison of nutritional measurements between the two groups

As shown in Supplementary Figure S2, before the intervention, there was no significant differences in body weight (Supplementary Figure S2A), triceps skinfold thickness (TSF) (Supplementary Figure S2B), and biomarkers [Total protein (TP) (Supplementary Figure S2C), Serum albumin (ALB) (g/L) (Supplementary Figure S2D), serum prealbumin (PA) (mg/L) (Supplementary Figure S2E)] between the intervention group and control group (*P* > 0.05). After the intervention, compared with the control group, the intervention group showed an increase in the serum levels of PA (*P* < 0.01) (Supplementary Figure S2E). However, there was no statistically significant difference between the intervention group and the control group in terms of body weight (Supplementary Figure S2A), TSF (Supplementary Figure S2B), TP (Supplementary Figure S2C) and ALB (Supplementary Figure S2D) (*P* > 0.05).

## 4. Discussion

The observation of similar improvements in swallow functional outcomes at 12 weeks post rehabilitation in the control group is also consistent with the findings of a systematic review of nine studies which estimated the impact of MDT based rehabilitation program among PSD patients (37). To explore this issue, MDT team could formulate standardized, individualized and comprehensive rehabilitation plans for PSD patients (38). At the same time, this research reflects the nature and extent of rehabilitation prescribed to PSD patients which were similar, with the main difference lying in the mode of rehabilitation delivery (tele-rehabilitation or not) (39). The difference in mode of service delivery highlights the strength of tele-rehabilitation which often facilitates more interactions between the service users (patients) and the service providers (physiotherapies). Although the swallowing function, quality of life, self-efficacy and social support index data of the two groups after the intervention are significantly improved compared with the baseline data, tele-rehabilitations overcome the barriers related to access to services caused by distance or difficulty of patient's mobility, potentially encouraging continuity of care and reducing the costs of the healthcare system (40).

In this research, after the smart health-based rehabilitation intervention, the WST and SAA scores of patients with PSD significantly decreased when compared with the control group (*P* < 0.05). This somewhat agreed with studies by Wu and Wang (41) and Gandolfi et al. (42). To explore this issue, the sample video of basic swallowing function rehabilitation training on the WeChat cloud platform could set an example for caregivers of PSD patients. Especially after discharge, the caregivers of PSD patients could repeatedly click the video to watch, which strengthens their proficiency in the rehabilitation of basic swallowing function. Videos on the WeChat cloud platform were classified according to the purpose of the swallow function exercise, which could help caregivers of PSD patients understand the benefits of each exercise on rehabilitation. At the same time, the WeChat punch in applet of daily monitoring report of rehabilitation exercises improved the rehabilitation adherence of caregivers of PSD patients in the implementation of basic swallowing function rehabilitation and ensured the rehabilitation training effect of oral–facial and laryngeal motor exercises and sensorial stimulation exercises (43). Moreover, the smart health-based rehabilitation intervention model could realize the interaction between neurology specialists, rehabilitation nurses, physiotherapists, nutritionists, caregivers of PSD patients and PSD patients through the WeChat cloud platform. Through one-to-one, face-to-face communication, the rehabilitation nurses, physiotherapists and nutritionists in the early stage helped the caregivers establish standardized operating procedures (44).

Enhancing patients' self-efficacy can increase their health status by improving health behavior and ultimately improve their quality of life (45). Bonetti et al. (46) conducted a follow-up survey on 203 patients with stroke after discharge and found that the level of self-efficacy was a strong predictor of the rehabilitation process of swallowing function. In this study, compared with the control group, the smart health-based rehabilitation significantly improved the score of SSEQ (*P* < 0.05). To explore this issue, one-on-one teaching of MDT members enables patients and their caregivers to gain direct experience. After PSD patients were discharged, MDT members continuously corrected the behavior of the caregivers to enhance the accuracy of their direct experience. Furthermore, in the peer education WeChat group, peer education was integrated into the rehabilitation training so that patients and caregivers were supplemented by alternative experience, and their self-efficacy was improved.

Quality of life describes an individual's overall wellbeing based on daily experience. The quality of life of patients with PSD usually decreases due to prolonged eating time, fatigue caused by poor nutritional status, decline in social support, psychological anxiety and depression (12). In this study, the smart health-based rehabilitation effectively enhanced the quality-of-life score of SWAL-QOL for PSD patients. On one hand, smart health-based rehabilitation can effectively improve the WST and SAA scores, enhance the swallowing function of patients, reduce the time of eating and finally improve the quality of life of patients. On the other hand, patients' fatigue in the dimension of quality-of-life scale is closely related to their poor nutritional status (47). Smart health-based rehabilitation can effectively improve the expression level of PA, reduce the patients' sense of fatigue, and finally improve the quality of life of PSD patients.

Malnutrition is very common in patients with PSD. At present, the malnutrition evaluation indexes of PSD include body weight, TSF, TP, ALB and PA. These measurements are recommended in the European Stroke Organization and European Society for Swallowing Disorders guidelines to monitor the nutritional status of PSD (30). Peng et al. (48) found that enteral nutritional suspension (TPF-FOS) JEVITY could effectively improve the expression of PA in stroke patients, but no statistical significance existed in weight, TSF, TP, ALB or other indicators compared with the control group (*P* > 0.05). In line with the results of Peng et al. (48), smart health-based rehabilitation can only improve the expression of PA in patients with PSD, and no significant difference existed in other nutritional indexes compared with the control group (*P* > 0.05). Body weight, TSF, TP and other indicators are not easy to change in a short time and are affected by oedema. Although ALB has always been the main index of nutritional risk assessment and prognosis in patients with PSD, its level cannot reflect the nutritional status of patients early and dynamically. However, the half-life of PA is only 2 days, and it can have significant changes in short-term protein supplementation (49). The continuous monitoring of PA levels can be used as a dynamic monitoring index of malnutrition.

This study has some limitations. The development time of smart health-based rehabilitation programs in China is relatively short. Moreover, smart health-based rehabilitation programs cannot be blind to implementers and patients, which may lead to performance bias. Furthermore, the intervention time is relatively short at only 12 weeks. Additionally, to control for initial spontaneous recovery, we included PSD patients with a minimum limit of 4 weeks by referring to the published literature (16, 50, 51). However, we still cannot completely rule out some positive effects of patients' spontaneous recovery on outcome measurements. Considering the medical ethical requirement, we could not include PSD patients with a minimum limit of 6–7 months. Moreover, we also could not set up a non-rehabilitation group to explore the outcome impact of PSD patients' spontaneous recovery rehabilitation. Furthermore, the National Institutes of Health Stroke Scale (NIHSS) is accepted as the definitive clinical examination to assess the neurological impairment severity of stroke patients (52). Conventional neuroradiological tools, such as CT and MRI, are useful to reveal radiological markers of neuropathology in PSD patients (53). However, due to the limitation of research funds, we did not collect data in this brief research study on the neurological impairment severity and neuroradiological markers independently associated with PSD outcomes. We would build the collection of these data into a further full-scale study.

## 5. Conclusion

Overall, our data revealed that smart health-based rehabilitation is significantly beneficial to the swallowing function, quality of life, self-efficacy, and social support for Chinese PSD patients when compared with routine rehabilitation. However, nutritional measurements were not significantly improved in such patients under the smart health-based rehabilitation when compared the routine rehabilitation. In the future, it is necessary to extend the intervention time to further evaluate the long-term efficacy of smart health-based rehabilitation on nutritional measurements of PSD patients in China.

## Data availability statement

The original contributions presented in the study are included in the article/Supplementary material, further inquiries can be directed to the corresponding author.

## Ethics statement

The studies involving human participants were reviewed and approved by Dongguan Houjie Hospital Affiliated to Guangdong Medical University. Written informed consent to participate in this study was provided by the participants' legal guardian/next of kin. Written informed consent was obtained from the individual(s), and minor(s)' legal guardian/next of kin, for the publication of any potentially identifiable images or data included in this article.

## Author contributions

J-RZ and Y-EW: conceptualization, data curation, and writing. Y-FH: software. S-QZ and W-LP: analysis. J-XH: validation. Q-PH: supervision and funding acquisition. All authors read and approved the final manuscript.
