# Supplementary material for: Effectiveness of smart health-based rehabilitation on patients with poststroke dysphagia: A brief research report
Source: Front Neurol. 2023 Jan 9;13:1110067. doi: 10.3389/fneur.2022.1110067 (PMC9868154; doi:10.3389/fneur.2022.1110067)
Supplement: Supplementary file 1 [file Data_Sheet_1.docx]

Supplementary Material

Effectiveness of Smart Health-based Rehabilitation on Patients with Poststroke Dysphagia: A Brief Research Report

Jian-Rong Zhang^1†^, Yu-E Wu^1†^, Yan-Fang Huang^1^, Shu-Qing Zhang^1^, Wen-Li Pan^1^, Jin-Xia Huang^1^, Qing-Ping Huang^1*^

***Correspondence:**Dr. Qing-Ping Huang, Department of Nursing, Dongguan Houjie Hospital Affiliated to Guangdong Medical University, Dongguan, Guangdong Province, 510000, China. Email: [hqphuli@sina.com](mailto:hqphuli@sina.com)

# Supplementary Figures and Tables

## Supplementary Figures


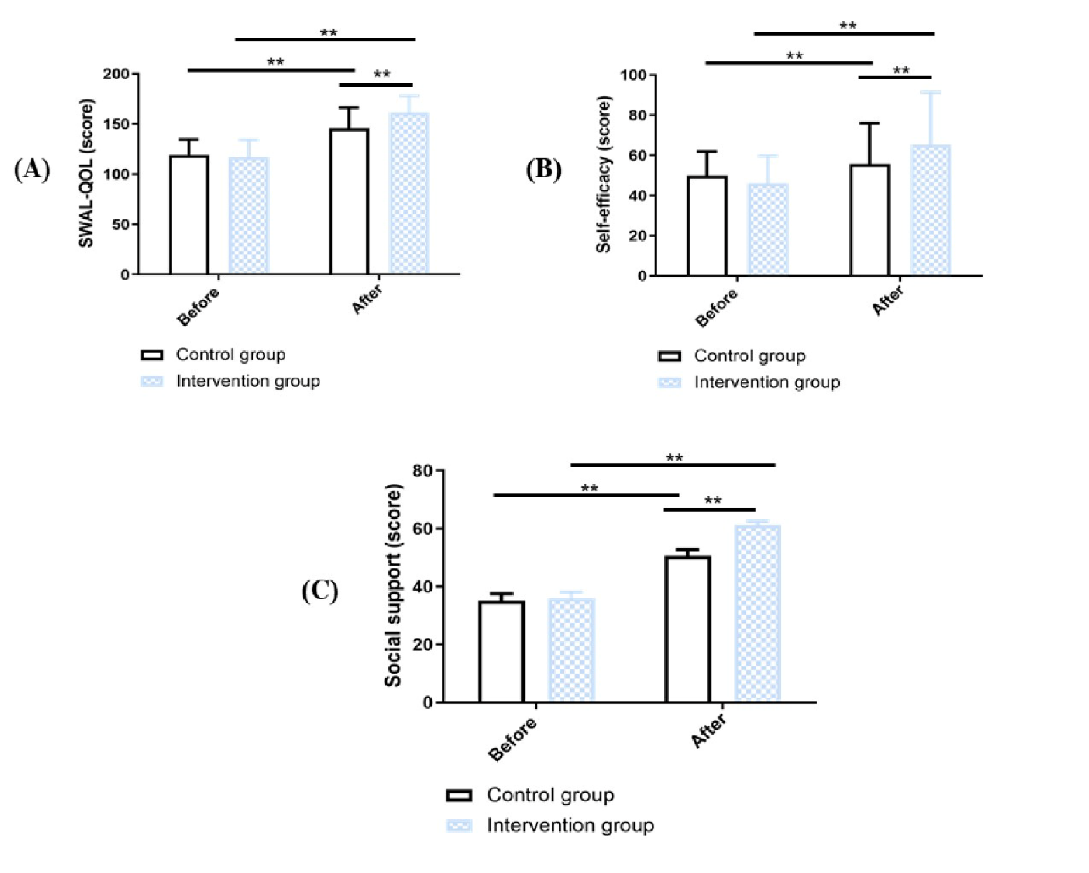


**Supplementary Figure S1**. Comparison of quality of life (A), self-efficacy (B) and social support (C) between the intervention and control group, ^**^*P*<0.01.


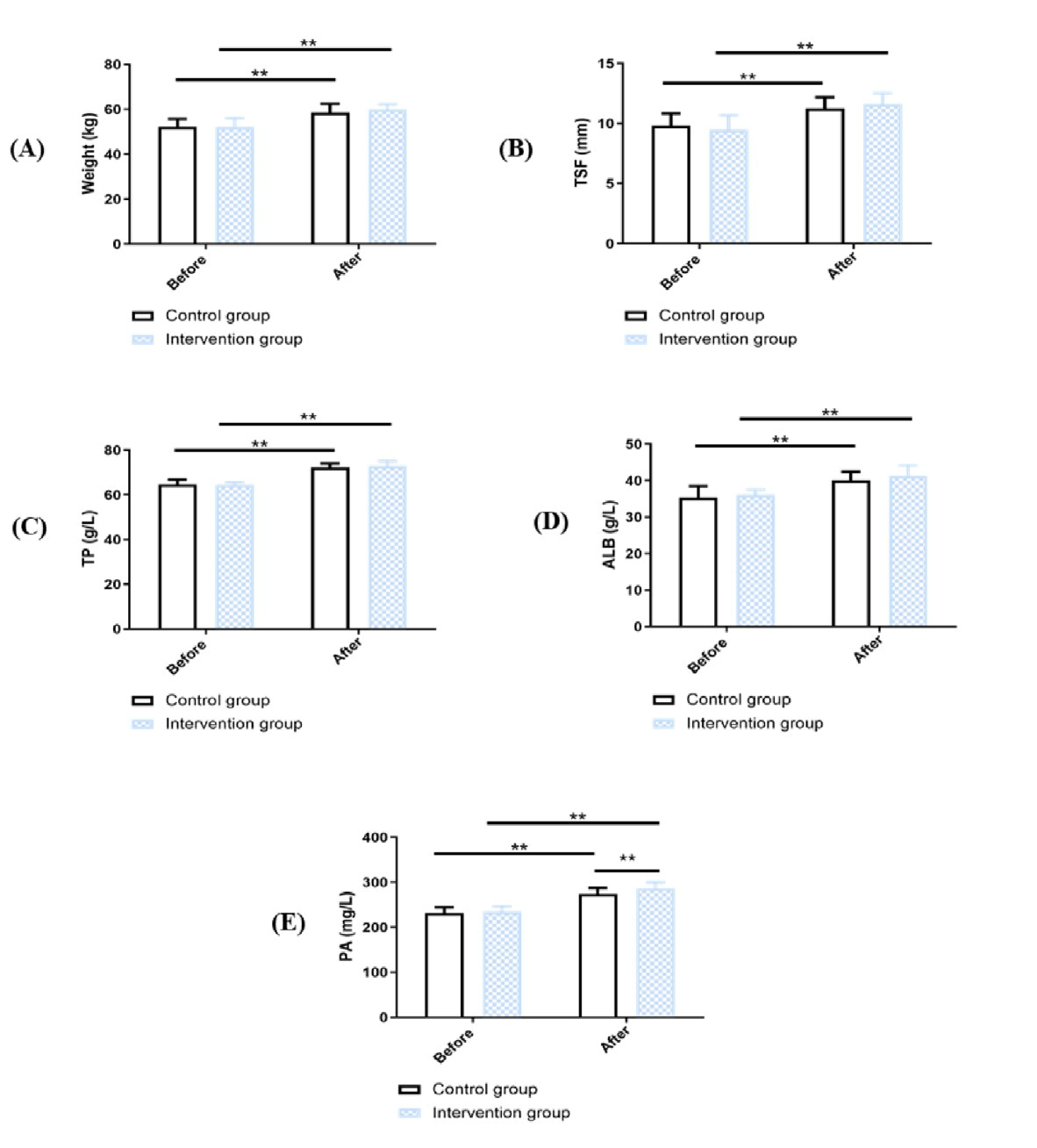


**Figure S2.** Comparison of Body weight (A), TSF (B), TP (C), ALB (D) and PA (E) between the intervention and control group before and after the intervention, ^**^*P*<0.01.
